# Computational investigation of conformational variability and allostery in cathepsin K and other related peptidases

**DOI:** 10.1371/journal.pone.0182387

**Published:** 2017-08-03

**Authors:** Marko Novinec

**Affiliations:** Department of Chemistry and Biochemistry, Faculty of Chemistry and Chemical Technology, University of Ljubljana, Ljubljana, Slovenia; University of Queensland, AUSTRALIA

## Abstract

Allosteric targeting is progressively gaining ground as a strategy in drug design. Its success, however, depends on our knowledge of the investigated system. In the case of the papain-like cysteine peptidase cathepsin K, a major obstacle in our understanding of allostery is represented by the lack of observable conformational change at the active site. This makes it difficult to understand how binding of effectors at known allosteric sites translates into modified enzyme activity. Herein, we address this issue by a computational approach based on experimental data. We analyze the conformational space of the papain-like family and the positioning of cathepsin K within it using principal component analysis and molecular dynamics simulations. We show that human cathepsin L-like endopeptidases (cathepsins L, K, S and V) adopt similar conformations which are distinct from their non-animal counterparts and other related peptidases. Molecular dynamics simulations show that the conformation of cathepsin K is influenced by known allosteric effectors, chondroitin sulfate and the small molecules NSC13345 and NSC94914. Importantly, all effectors affect the geometry of the active site around sites S1 and S2 that represent the narrowest part of the active site cleft and the major specificity determinant in papain-like endopeptidases. The effectors act by stabilizing pre-existing conformational states according to a two-state model and thereby facilitate or hinder the binding of substrate into the active site, as shown by molecular docking simulations. Comparison with other related enzymes shows that similar conformational variability and, by implication, allostery also exist in other papain-like endopeptidases.

## Introduction

The extent of conformational change associated with allosteric regulation ranges from movement of entire domains to minimal changes in the protein backbone [1]. While cases involving significant changes in protein structure are often nearly self-explanatory at the structural level, subtle structural alterations are more delicate to capture and interpret [2]. They may involve alterations in pre-existing equilibria of functionally distinct conformational states [3] and complex ligand-binding mechanisms involving conformational selection or induced fit [4]. Moreover, it is increasingly recognized that structural characterization of small conformational changes may be hampered by experimental restrictions such as the conditions used to grow protein crystals which are frequently far from physiological and enforce a particular conformational state on the studied protein [2].

The cysteine peptidase cathepsin K is becoming established as a model enzyme for regulation of papain-like peptidases via sites distant from the active site. However, lack of significant conformational change in X-ray structures has thus far hindered our understanding of this system. The enzyme itself is a critical enzyme in bone homeostasis [5] and has potent collagenase activity [6]. Its targeting is considered one of the most promising approaches for future treatment of osteoporosis [7]. Unfortunately, the first generation of orthosteric inhibitors had limited success. The most successful compound odanacatib [8] concluded phase 3 clinical trials [9] but was terminated recently due to side effects. With regards to allostery, cathepsin K is the first member of the papain-like family in which this mode of regulation was characterized [10]. Glycosaminoglycans (GAGs) were the first known allosteric effectors of cathepsin K with presumably major biological roles in the regulation of its collagenase activity [11]. Chondroitin-4-sulfate (C4S) specifically is a major enhancer of collagen degradation by cathepsin K [11]. Current evidence suggests that the biologically active forms involve oligomeric cathepsin K/C4S complexes with varying stoichiometry and collagenase activity. Thus far, three binding sites for C4S are known on cathepsin K [12, 13]. Other GAGs were also attributed roles in collagenolysis regulation with effects ranging from activation to inhibition [13, 14]. GAGs were also shown to increase the activity of cathepsin K on synthetic substrates and protein substrates other than collagen, e.g. elastin, and are thus allosteric effectors of cathepsin K in general [10].

In our work, we have been investigating allosteric mechanisms by combining computational and experimental approaches. Using the statistical coupling analysis we identified the protein sector of papain-like peptidases [15], an evolutionarily conserved network of residues that transmits allosteric communication [16]. Based on these results we identified a novel allosteric site on cathepsin K [15] which was later shown to partially overlap with a C4S-binding site [13]. Up-to-date we have characterized two effectors that bind at this site (NSC13345 and NSC94914, respectively, both from the US NCI Developmental Therapeutics Program). Both had inhibitory activity on the hydrolysis of synthetic and protein substrates, but different activity profiles, presumably due to their different modes of interaction with the allosteric site [15, 17].

Despite these data, the acceptance of cathepsin K as a model for allosteric regulation in papain-like peptidases has been hindered due to the absence of conformational change in X-ray structures of cathepsin K in complexes with allosteric effectors and orthosteric inhibitors. Herein we address this issue by a computational approach based on the significant amount of available structural data. Up-to-date over 50 X-ray structures of cathepsin K alone and over 400 structures of papain-like peptidases are available from the Protein Data Bank, hence a large enough sample is available for thorough analysis. We investigate the conformational space of the papain-like family, the position of cathepsin K within it, we examine the conformational variability of the molecule alone and in complexes with known effectors and simulate the binding of a peptide substrate into the active site in the presence of different effectors. Based on these results we provide a molecular model for allosteric regulation of cathepsin K and provide an outlook of similar mechanisms in other related enzymes for which experimental data are not yet available.

## Results and discussion

### Conformational space of papain-like peptidases

**Fig 1:**
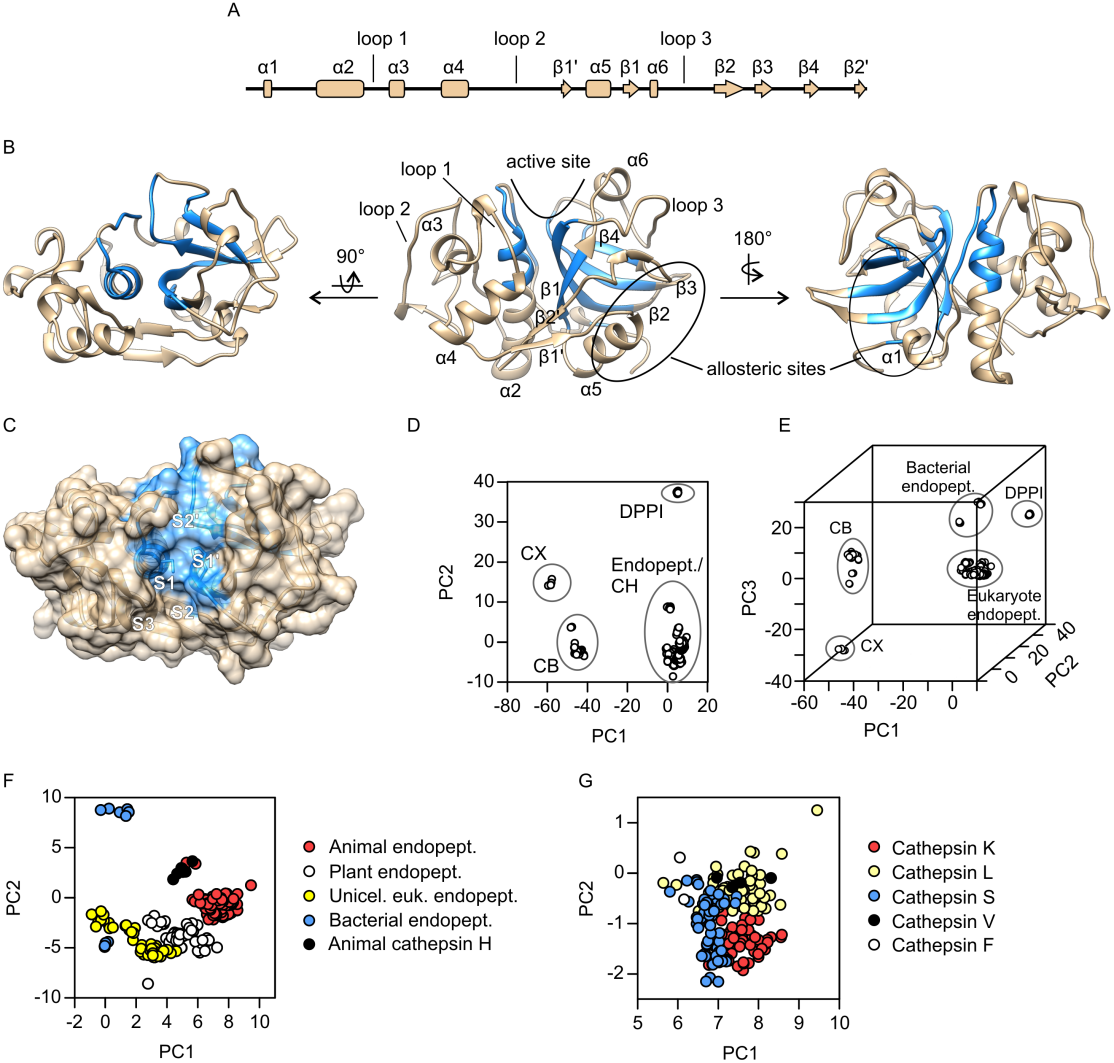
Overall structure and principal component analysis of papain-like peptidases. The analysis is based on a sample of 372 non-redundant structures retrieved from the Protein Data Bank (S1 Table). (A) Diagram of secondary structure elements along the sequence of cathepsin K. (B) Invariant core of the family (shown in blue) mapped on the structure of cathepsin K (PDB accession code 1ATK). The molecule is shown in the standard orientation (center), from the back side, i.e. rotated by 180° around the vertical axis (right) and from the top side, i.e. rotated by 90° around the horizontal axis (left). The arrow indicates the location of the active site cleft and the locations of two allosteric sites are circled. (C) Top view of the active site cleft shown in surface representation. Invariant core residues are colored blue. Individual substrate-binding sites within the cleft are marked S3 through S2'. The catalytic diad Cys-His is shown as sticks. (D) Conformational space of papain-like peptidases represented in a two-dimensional plot of the first two PCs. (E) Conformational space of papain-like peptidases represented in a three-dimensional plot of the first three PCs. (F) and (G) Progressively zoomed views of portions of the conformational space comprising all papain-like endopeptidases (panel F) and animal papain-like endopeptidases (panel G), respectively. The plots in panels D, F and G were drawn with GRAPHPAD PRISM 5.0 software (GraphPad Software, La Jolla, CA, USA) and the plot in panel E was drawn in the R environment. Molecular graphics were prepared with UCSF CHIMERA software [36]. Abbreviations: CB—cathepsin B, CH—cathepsin H, CX—Cathepsin X, DPPI—dipeptidyl-peptidase I (cathepsin C), endopept.–endopeptidase, euk.–eukaryote, unicel.–unicellular.

As the starting point of this work, we aimed to obtain a perspective of the total conformational variability within the papain-like family. For this purpose we investigated its conformational space using principal component analysis (PCA) on the complete ensemble of X-ray structures currently available from the Protein Data Bank (see S1 Table for a complete list of PDB entries). As the major focus of this work, the cathepsin K molecule was used as reference for data analysis and presentation. Fig 1A shows the distribution of secondary structure elements along the sequence of human cathepsin K with designations that will be used throughout the manuscript. The three consecutively numbered loops will also be discussed in detail in the continuation.

In the first step of the analysis, the invariant core, which comprises residues with highly conserved spatial positions at the family level, was calculated and mapped on the structure of human cathepsin K, as shown in Fig 1B (invariant core residues are colored blue). The enzyme itself has the typical papain-like endopeptidase fold consisting of L- and R-subdomains according to their relative positions in the standard orientation (middle representation in Fig 1B). The active site cleft is positioned between both subdomains at the top of the molecule. Each subdomain contributes one residue to the catalytic diad Cys-His and loops lining the cleft at the respective sides. The L-subdomain contains a central α-helix (α2) which runs vertically across the whole molecule and two shorter α-helices (α3 and α4), whereas the R-subdomain contains a central antiparallel four-stranded β-sheet with a barrel-like organization (β-strands 1 through 4) and two short α-helices (α5 and α6). Both subdomains are connected via a two-stranded antiparallel β-sheet at the front (β-strands 1' and 2') and the N-terminal loop at the back of the molecule which contains a short α-helical segment (α1). The invariant core of the molecule consists of the N-terminal portion of the helix α2, the four-stranded β-sheet, a portion of the loop connecting both subdomains on the back side and a single residue (position 6 in cathepsin K) immediately preceding helix α1. Function-wise, the invariant core in cathepsin K comprises the active site and the C4S-binding site on the back side, and lies beneath the allosteric site at the bottom right side which is bound by both GAGs and small molecule effectors. Within the active site cleft (Fig 1C), the invariant core comprises the S1 site (according to the nomenclature by Schechter and Berger [18]) as well as the primed sites, but only part of the S2 site, which is the primary specificity determinant in papain-like endopeptidases. Sites preceding the S2 site are not formed by spatially conserved residues, hence this part is more diverse between family members and potentially more flexible within individual molecules.

For the purposes of PCA, the ensemble was superposed onto the invariant core prior to analysis. The results of PCA showed that most of the conformational variability (approx. 64%) is contained within the first two principal components (PC1 and PC2) which account for 50.4% and 13.5% of sample variance, respectively, enabling its representation in a straightforward two-dimensional diagram. The plot of PC1 versus PC2 (Fig 1D) shows the existence of four groups within the papain-like family that are distinguished by the positions of cleavage sites within protein substrates. The exopeptidases cathepsin B (a peptidyl dipeptidase with additional pH-dependent endoproteolytic activity), cathepsin X (a carboxypeptidase) and dipeptidyl-peptidase I (also known as cathepsin C) each form separate groups, whereas papain-like endopeptidases form the fourth group. This basic division reflects the divergence of these groups from the common ancestor in early eukaryotes [19]. The endopeptidase group also includes the aminopeptidase cathepsin H which, based on proenzyme homology, is more closely related to the endopeptidases than other exopeptidases [19]. Addition of PC3 (7% of sample variance) results in separation of bacterial and eukaryotic endopeptidases (Fig 1E), but apart from this does not provide significant additional information. Therefore, the plot of PC1 versus PC2 will be used for analytical purposes in the continuation. The endopeptidase group itself shows well-structured hierarchy based roughly on the phylogenetic relationships of the proteins (Fig 1F). Bacterial proteases form two distinctive groups. The lower one is formed by xyllelain, a papain-like peptidase from pathogenic strains of the plant pathogen *Xyllela fastidiosa* [20], whereas the upper group is formed by the *Clostridium* peptidase Cwp84, an S layer multidomain protein [21]. Distinctive groups are also formed by endopeptidases from unicellular eukaryotes (mostly *Trypanosoma* and *Plasmodium* species), plants and animals. Within the animal group, which contains mostly mammalian enzymes, cathepsin H lies separate from the rest, whereas the endopeptidases are clustered together, with the exception of two structures of procathepsin L2 from the mealworm *Tenebrio molitor* which cluster with cathepsin H. Within the endpopeptidase group (Fig 1G), cathepsins L, S and K form partially overlapping clusters and cathepsin V is clustered together with cathepsin L. Cathepsin F is located outside of the main clusters, as expected due to its distant evolutionary relation [22], but nevertheless overlaps with some cathepsin L and S structures, thus reflecting a similar overall conformation of the molecule. Further subdivision can be observed within the plant and protist groups, but will not be further investigated herein.

**Fig 2:**
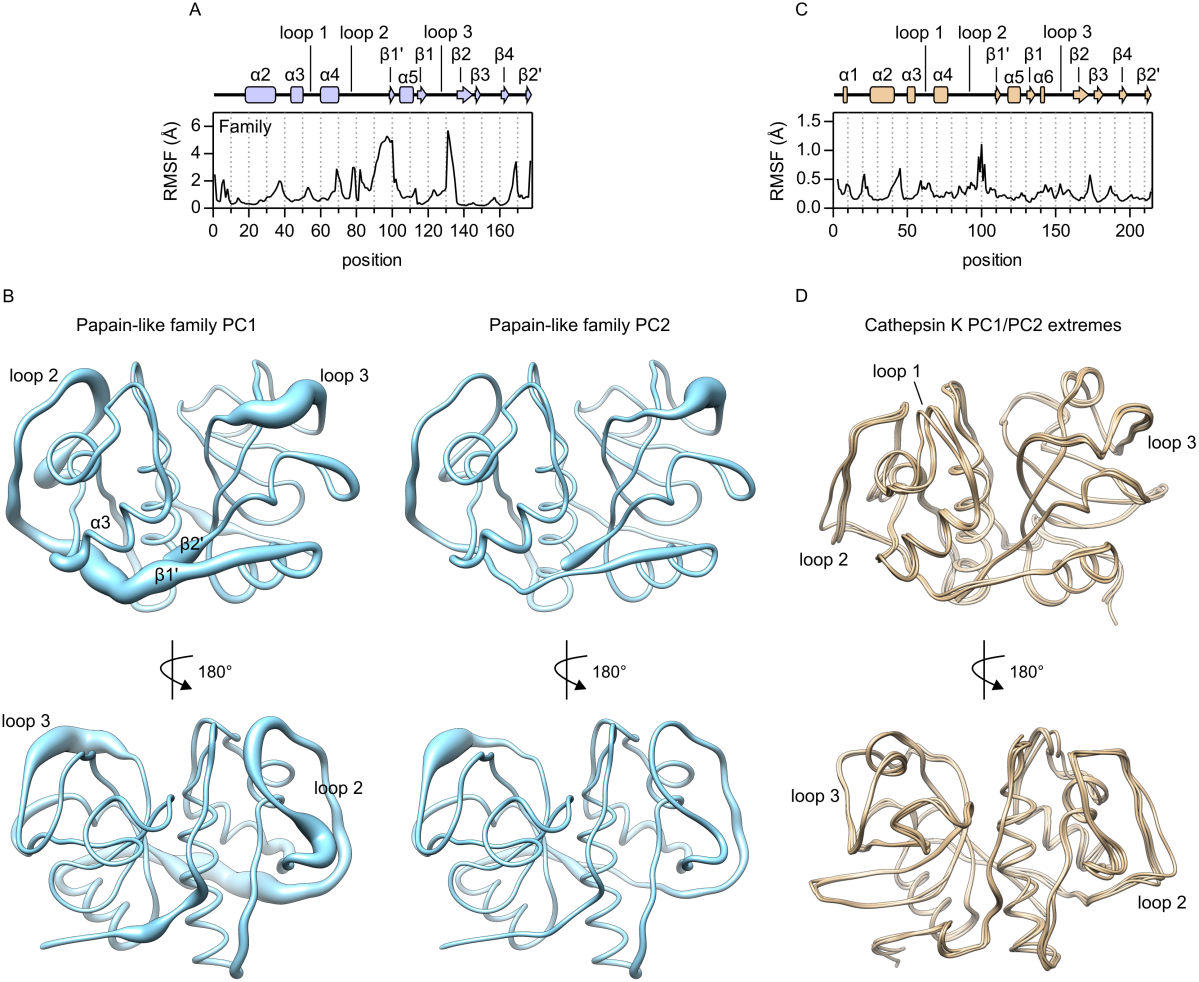
Conformational variability of papain-like peptidases. (A) Root mean square fluctuations (RMSF) at 178 non-gap positions in the sequence alignment of the ensemble used for PCA. The diagram of secondary structure elements is aligned with the positions in the plot and the numbering of elements corresponds to Fig 1A. (B) Conformational variability contained within PC1 (left) and PC2 (right) shown in worm representation of a hypothetical protein consisting of 178 non-gap positions in the sequence alignment of the ensemble. (C) Root mean square fluctuations (RMSF) in cathepsin K. The analysis was performed on a sample of 55 non-redundant cathepsin K structures. The diagram of secondary structure elements is aligned with the positions in the plot. (D) Superposition of cathepsin K structures with extreme values of the first two PCs (PDB accession codes 1BY8, 1U9W, 1YK7 and 3OVZ). The plots in panels A and C were drawn with GRAPHPAD PRISM 5.0 software (GraphPad Software, La Jolla, CA, USA). Molecular graphics were prepared with UCSF CHIMERA software [35].

Structural variability within the family, as identified by PCA, is shown in Fig 2A and 2B. The first shows root mean square fluctuations (RMS fluctuations, RMSF) along the sequence alignment of the ensemble and the second shows three-dimensional representations of the variance explained by the first two PCs. The most variable regions include the long left-side loop connecting helix α4 and strand β1' (loop 2), strands β1' and β2', and the C-terminal part of the loop connecting strands β1 and β2 (loop 3) which lines the right side of sites S1 and S2. RMSF values for these regions are between 2 Å and 5 Å (Fig 2A). Structural superposition of various papain-like peptidases shows that these fluctuations are the result of structural differences between family members. For additional insight into their structural diversity, three-dimensional structures of representative family members are shown in S1 Fig and their superpositions with cathepsin K in S2 Fig. It must also be noted that the analysis in Fig 2A and 2B is based only on 178 non-gap positions in the sequence alignment of the ensemble and does not account for additional structural elements found only in individual members of the family. Interestingly, the corresponding regions with high fluctuations are also not part of the previously identified protein sector of papain-like peptidases [15]. Thus, they appear to represent elements that evolve independently from the rest of the molecule.

The results of PCA also showed dispersion of PC values calculated for the same protein, indicating structural variabilility of the associated regions within individual proteins. To analyze the variability of cathepsin K, we examined RMS fluctuations in 55 non-redundant cathepsin K structures (Fig 2C) and created a superposition of four structures with extreme values of PC1 and PC2, respectively (Fig 2D). In general, little variability was observed in in the molecule. Loop 2 (residues 91 through 105 in cathepsin K) was the most variable part, however, maximal RMSF values were only about 1 Å. This is well below the 2 Å threshold, which is a common criterion for conformational change [1]. Nevertheless, this region appears to be functionally important in cathepsin K, as it contains two predicted allosteric sites that are presumably bound by several different effectors [15, 23, 24] and is also involved in the proposed oligomerization of cathepsin K [13]. Other parts that were identified as flexible at the family level showed only minor fluctuations, which could also be considered as artefacts from the modeling of loop regions in the X-ray structures. Interestingly, structural superposition (Fig 2D) also showed a different conformation of loop 1 in one of the molecules. This loop lines the left side of sites S1, S2 and S3 and showed comparably little variance at the family level (Fig 2A and 2B). The observed conformation belongs to the structure of procathepsin K (Fig 2D shows the structure available under PDB accession code 1BY8 [25]) and was thus far not observed in mature cathepsin K. Nevertheless, it indicates that loop 1 can adopt different conformations.

Thus, taken together, the results of PCA indicate that the loops surrounding the non-primed sites of the active site (loops 1 and 3) are flexible, which may be important for the functional properties of cathepsin K and other papain-like peptidases. However, at least in the case of cathepsin K, no convincing evidence of conformational variability could be obtained from X-ray data. Lack of observable change could be due to crystal contacts or to crystallization conditions that usually involve high salt concentrations, to which cathepsin K is known to be sensitive [10]. Since most structures include inhibitors bound into the active site, these could also influence the conformation of the latter.

For further insight into the conformational fluctuations in cathepsin K, the ensemble of its X-ray structures was also analyzed by normal mode analysis. Fluctuations corresponding to the first five (non-trivial) normal modes are illustrated in S3 Fig and the corresponding coordinates are supplied as S1 Dataset. Essentially, residues that comprise the invariant core show little fluctuation, whereas the rest of the molecule is more flexible. Normal mode 1 shows coordinated movement that comprised most of the molecule. Most importantly, it comprises loops surrounding the active site as well as structural elements comprising the allosteric sites (see Fig 1B for reference) indicating that intramolecular movements are coupled between these sites.

### Conformational space of cathepsin K investigated by molecular dynamics (MD) simulations

Results in the previous section showed that conformational variability exists in the family of papain-like peptidases, but only limited variability could be observed in cathepsin K by crystallographic analyses. To investigate the conformational space not reachable by X-ray crystallography, we performed MD simulations of cathepsin K alone and in complexes with substrate or allosteric effectors. For comparison, the analysis was also extended to other animal endopeptidases in their unbound states. The calculated trajectories were analyzed using PCA and mapped onto the conformational space defined by the X-ray ensemble in the previous section. Overall, trajectories contained between 100 and 240 ns of total simulation time (see Methods section MD simulations for details) and each run was repeated multiple times to minimize the impact of random events.

**Fig 3:**
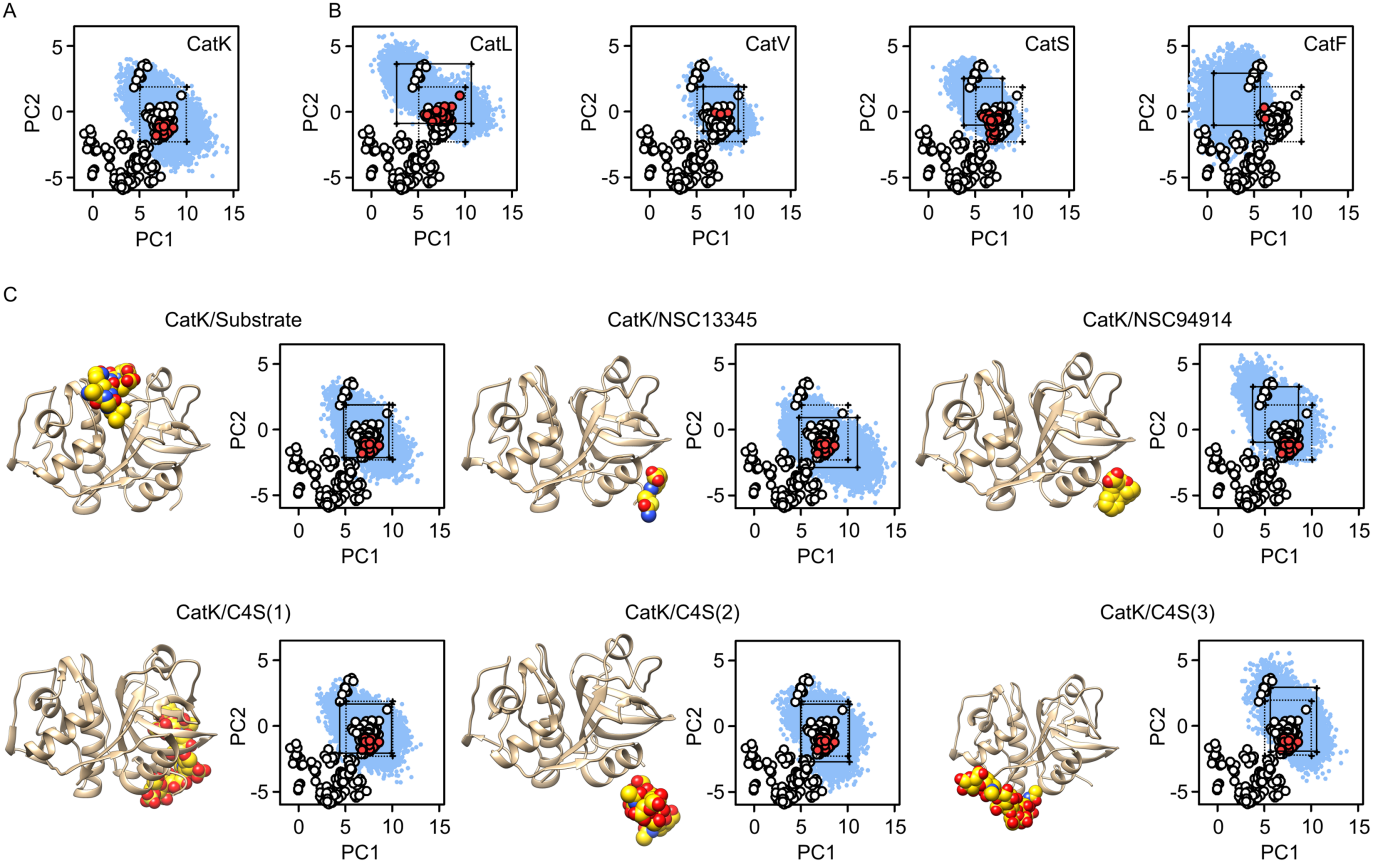
Principal component analysis of molecular dynamics simulations. (A) Projection of MD conformers of cathepsin K onto the PC1/PC2 plane of the conformational space defined by the X-ray ensemble (see Fig 1). Only the section of interest is shown. The positions of animal peptidases from the X-ray ensemble are shown as red dots and non-animal peptidases as white dots, respectively. The positions of MD conformers are shown as blue dots. The rectangle denotes the boundaries of the 5th and 95th percentiles of each PC. (B) Projections of MD conformers of human cathepsins L, V, S and F onto PC1/PC2 plane of the conformational space defined by the X-ray ensemble. Full rectangles denote 5th and 95th percentiles of each PC for the examined MD ensemble, whereas dotted rectangles represent the boundaries of cathepsin K (see panel A). (C) Projections of MD conformers of cathepsin K in complexes with the substrate AGLKEDDA, small molecule effectors NSC13345 and NSC94914, and C4S bound to each of the three known binding sites onto the conformational space. Rectangles define the 5th and 95th percentiles of PC values as in panel B. The plots were drawn with GRAPHPAD PRISM 5.0 software (GraphPad Software, La Jolla, CA, USA) and molecular graphics with UCSF CHIMERA software [35].

As expected, MD conformers of cathepsin K sampled a significantly greater portion of the conformational space than observable by X-ray crystallography (Fig 3A). Its conformational space spans all X-ray conformers of animal endopeptidases as well as cathepsin H, but does not extend into the conformational space of its non-animal (i.e. plant) counterparts. To filter out low-populated states and for ease of comparison, the 5th and 95th percentiles were calculated for each PC and are represented graphically as a rectangle. The latter, therefore, excludes conformers with extreme values of either or both principal components and shows that the region of conformational space which includes between 80% and 90% of the »common« conformers is significantly smaller than the entire sampled conformational space. Nevertheless, in the case of cathepsin K, it still encompasses the entire X-ray ensemble of vertebrate endopeptidases, and, conversely, X-ray conformers of cathepsin K still represent only a small portion of the area.

Due to their structural and functional similarity to cathepsin K, all human papain-like endopeptidases (cathepsins L, S, V and F) were investigated by MD simulations in the same manner. Similar to the former, all enzymes sampled a significantly greater portion of conformational space in comparison to their X-ray structures (Fig 3B). Comparison of the 5th/95th percentile boundaries of both PCs showed significant overlaps of the conformational spaces between the evolutionarily closely related cathepsins K, L, S and V, indicating that all vertebrate cathepsin L-like enzymes adopt similar conformations. In contrast, the conformational space of the evolutionarily distant cathepsin F [22] was distinctively different from these peptidases.

In the continuation, we analyzed MD simulations of cathepsin K in complexes with different ligands. These included the octapeptide substrate AGLKEDDA bound into the active site to simulate the bound mode of the enzyme, allosteric effectors NSC13345 and NSC94914, and C4S bound to each of the three known binding sites on cathepsin K (Fig 3C). The particular substrate was chosen based on individual site preferences of human cathepsin K as determined by proteomic analyses [26, 27]. Cathepsin K/C4S complexes were investigated at 1:1 stoichiometry. The proposed oligomeric forms observed in X-ray structures [12, 13] were not included since their structure-function relationship is not fully understood. The results showed that MD conformers from these simulations were constrained to approximately the same conformational space as ligand-free cathepsin K. However, examining the 5th/95th percentile boundaries of PC values showed notable differences between free enzyme and its complexes with NSC13345 and NSC94914, respectively. These results indicate a redistribution of populations within the total conformational space accessible to cathepsin K. A similar effect was observed for C4S bound to the third binding site (complex 3), but not for the remaining simulations. Altogether, the observed differences were not drastic and the common conformers (i.e. rectangles) still overlapped significantly. Nonetheless, the observed differences are, for example, comparable to those between cathepsins K and S, i.e. two different peptidases. It is also of note that effectors NSC13345 and NSC9414 both caused redistribution of conformers, but in different directions in conformational space. Thus, despite binding to the same site, they exhibited different effects on protein conformation. Moreover, binding of C4S to the same site (complex 2) did not significantly affect the conformational space of cathepsin K. Altoghether, these results highlight our previous findings that allosteric effects triggered by the effectors strongly depend on their mode of interaction with this binding site [17].

**Fig 4:**
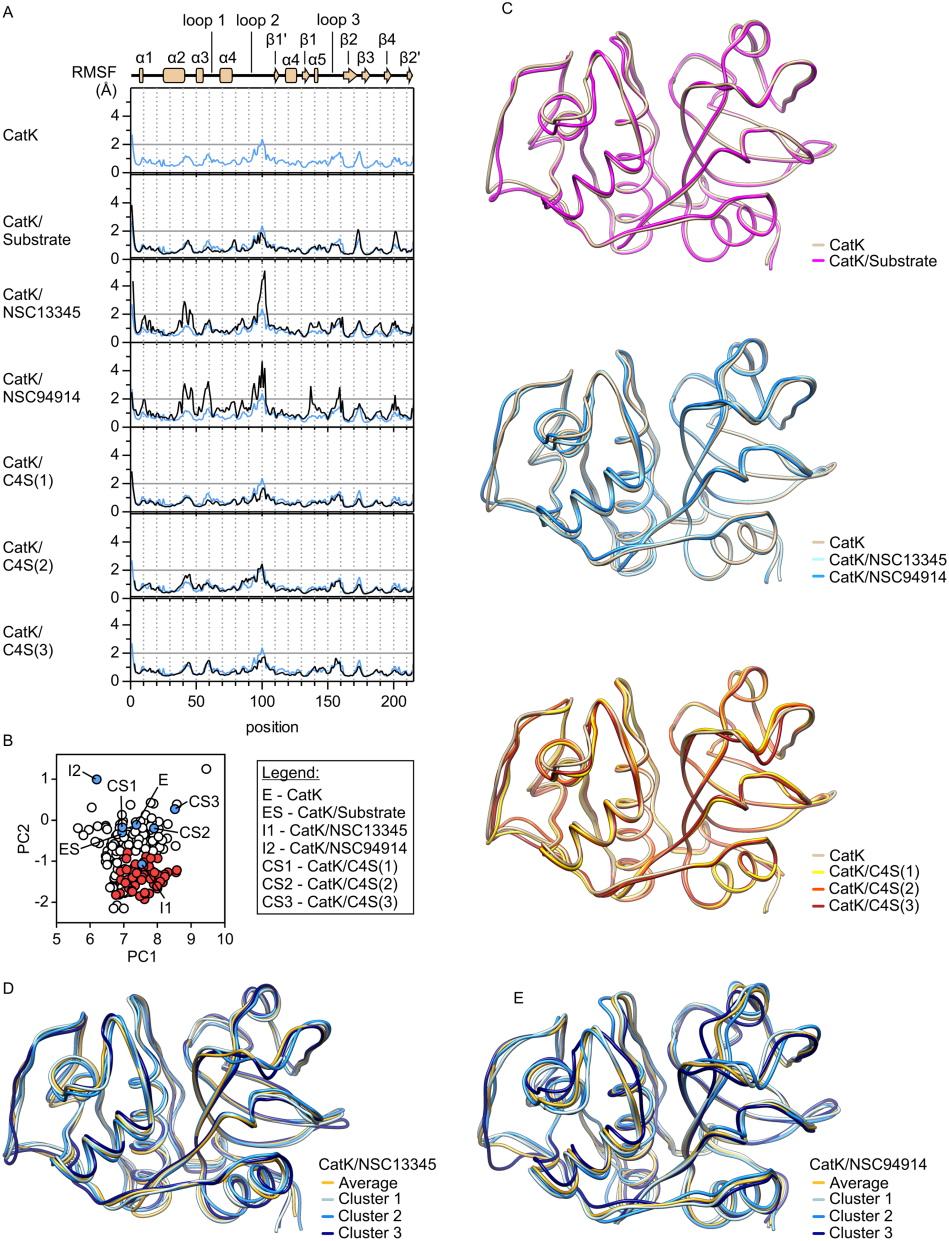
Structural analysis of molecular dynamics simulations. (A) Root mean square fluctuations (RMSF) in MD simulations of cathepsin K alone and in complexes with the substrate AGLKEDDA, small molecule effectors NSC13345 and NSC94914, and chondroitin sulfate bound to each of the three known binding sites. The diagram of secondary structure elements in cathepsin K is aligned with the positions in the plot. (B) Projections of average MD conformers of cathepsin K from the simulations in Fig 3 onto the conformational space. (C) Superpositions of average MD conformers of cathepsin K. Ligand-free cathepsin K is colored tan, substrate-bound enzyme is colored magenta, complexes with small molecule effectors NSC13345 and NSC94914 are colored light and dark blue, respectively, and C4S complexes 1, 2 and 3 (numbering according to Fig 3C) are colored yellow, orange and red, respectively. (D) and (E) Superpositions of average MD conformers and representative members of three largest conformer clusters identified in MD simulations of cathepsin K in complexes with NSC13345 (panel D) and NSC94914 (panel E), respectively. The plots were drawn with GRAPHPAD PRISM 5.0 software (GraphPad Software, La Jolla, CA, USA) and molecular graphics with UCSF CHIMERA software [36].

For structural evaluation of the observed differences in conformational space, RMS fluctuations were first calculated for each simulation (Fig 4A). As expected, fluctuations in ligand-free cathepsin K were higher than those determined for the crystal structures. However, RMSF values at most positions were still well below 2 Å and the mean RMSF value for the whole protein was about 0.8 Å, indicating the presence of a single conformation. The presence of substrate or C4S did not have significant effects on fluctuations in cathepsin K, whereas both small molecule effectors significantly increased the flexibility of the protein (mean RMSF values of 1.2 Å and 1.3 Å for NSC13345 and NSC94914, respectively), mostly due to increased flexibility of loop regions. Interestingly, the strongest effect was observed for loop 2, despite its location on the opposite side of the molecule with regards to the ligand-binding site.

For direct comparison between the simulations and to X-ray data, average MD conformers were calculated from each trajectory. Their positions within the conformational space are shown in Fig 4B and mirror the observations made in the previous paragraphs. Most average conformers lie outside of the conformational space accessible in crystallographic experiments, and complexes with both small molecule effectors lie farthest from ligand-free enzyme. Superposition of average MD conformers (Fig 4C) showed a potentiation of the differences observed by superposition of X-ray structures (see Fig 2D for comparison), but significant conformational changes were not observed. Highest degree of variability was associated with loops 1 and 2, and, to a smaller extent, loop 3.

In addition to the comparison of average MD conformers, we also attempted to identify individual conformations in the simulations of cathepsin K in complexes with NSC13345 and NSC94914, respectively, which had RMSF values significantly higher than 2 Å (Fig 4A) at multiple locations. In each case, three major clusters were identified. They contained 53% (cluster 1), 33% (cluster 2) and 6% (cluster 3) of all frames in the case of the NSC13345 complex and 59% (cluster 1), 28% (cluster 2) and 7% (cluster 3) in the case of the NSC94914 complex. Superpositions of representative frames from each cluster and average MD conformers are shown in Fig 4D and 4E for the NSC13345 and NSC94914 complexes, respectively. In general, the clusters differ among each other mostly by the conformations of loops 1, 2 and 3, and the differences are more pronounced in the NSC94914 complex. In both cases, however, the observed differences were small and the clusters were very sensitive to changes in calculation parameters. Therefore, average MD conformers were used for structural analysis in the continuation. Taken together, MD simulations showed that loop regions, especially loops marked 1, 2 and 3, are the only regions of significant conformational variability in cathepsin K. Therefore, the observable allosteric effects are likely to be transmitted via these loops.

### Conformational variability of the active site

**Fig 5:**
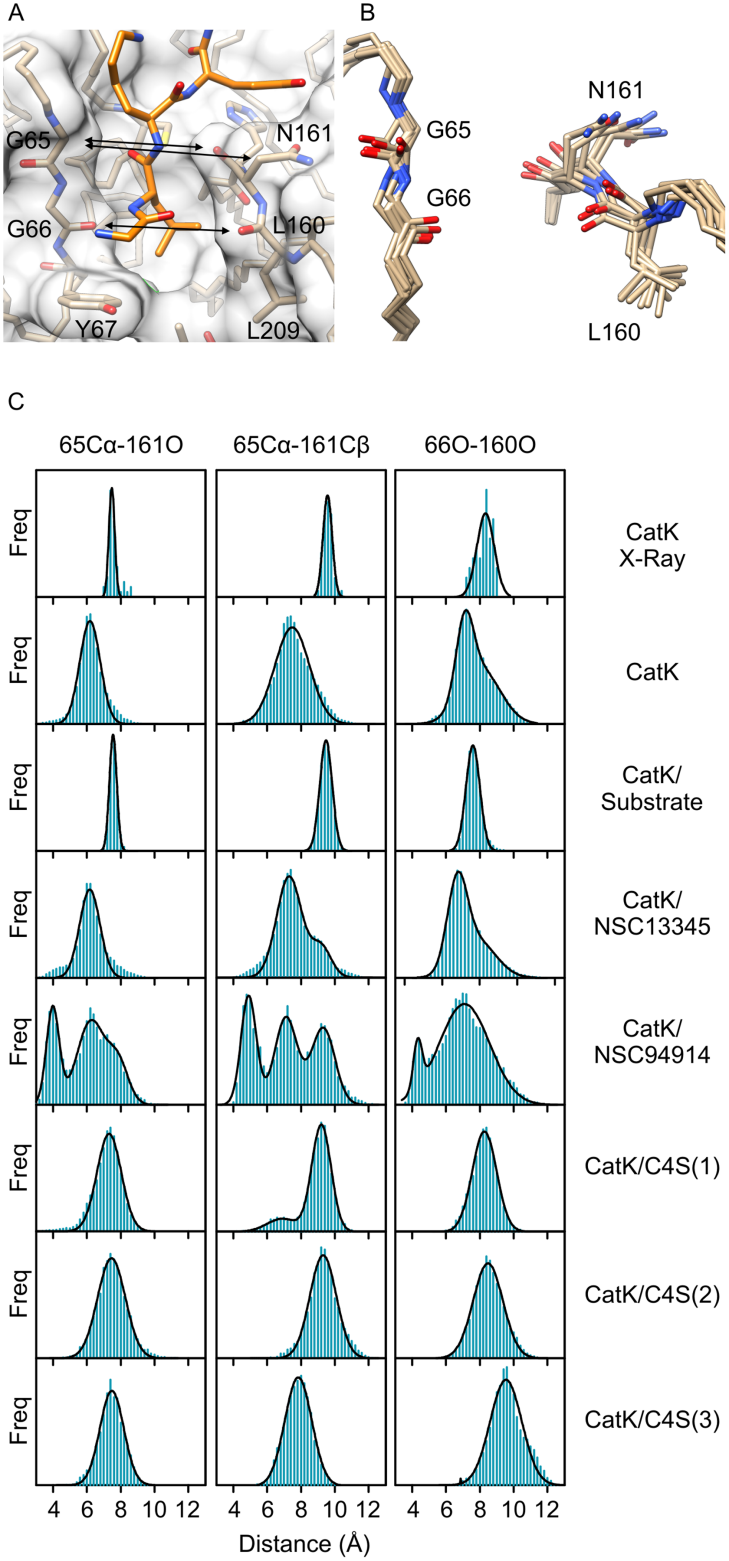
Active site geometry and dynamics in cathepsin K. (A) S1-S2 region of cathepsin K (shown as transparent surface and tan sticks) with a docked substrate molecule (orange sticks). Interatomic distances limiting the width of the S2-S1 cleft are marked by arrows. (B) Superposition of average MD conformers of cathepsin K in free form or in complexes with the substrate AGLKEDDA, C4S (all three binding modes), NSC13345 or NSC94914, respectively. The representation highlights the variability of the region. (C) Distribution of interatomic distances in MD simulations of cathepsin K in free form, substrate-bound form and in complexes with allosteric effectors, respectively. The distribution of distances in all non-redundant X-ray structures is given for comparison. All populations were segregated into 0.2 Å wide bins. The curves were fitted assuming normal distribution of interatomic distances using models assuming one, two or three populations, as appropriate. The analyses were performed with Graph Pad Prism 5.0 Software. Molecular graphics were prepared with UCSF CHIMERA software [36].

Ultimately, the action of effectors results in structural change of the active site and altered enzyme activity. The results in the previous sections indicate that only minor conformational changes occur in cathepsin K, however, these can be sufficient to modify enzyme activity, especially when keeping in mind that modulation of cathepsin K usually involves a few-fold decrease or increase in enzyme activity and not full inhibition or activation [10, 15, 17, 24]. We investigated the variability of the entire active site region in detail by comparing the distributions of interatomic distances that define its geometry between the MD simulations. Significant differences were observed only around sites S1 and S2 (Fig 5A) that are formed by loops 1 and 3, which were both identified as flexible regions in the previous sections. The remaining interatomic distances along the active site cleft did not vary significantly between simulations. Specifically, distances between residue pairs Gly65-Asn161 and Gly66-Leu160 were identified as the critical determinants. These two pairs form the narrowest part of the active site cleft and line both sides of the S2 site, the primary specificity determinant of papain-like endopeptidases. To avoid ambiguity, the pairs will be referred to as the proximal and distal pairs, respectively, based on their proximity to the catalytic diad and the region will be referred to as the S2-S1 cleft. Of the involved residues, Asn161 is the most flexible (Fig 5B). Main chain rotations result in its Cβ atom being oriented either away or towards the cleft, whereas rotation about the Cα-Cβ bond results in varying positions of its terminal amide group. For the purpose of comparison between simulations, we determined interatomic distances between the carbonyl O atom or the Cβ atom of Asn161 and the Cα atom of Gly65 which is the closest atom on the other side of the cleft. Analogously, we examined the distance between carbonyl oxygens of residues Gly66 and Leu160. The distributions of interatomic distances in MD simulations are shown in Fig 5C and Table 1. Data for the investigated ensemble of X-ray structures are also included for comparison. Taken together, the results showed that binding of substrate reduced the fluctuation of the enzyme and stabilized a particular conformation which can be assumed to be the catalytically productive conformation. Interatomic distances between each atom pair in the cathepsin K/substrate complex were similar to those determined for the X-ray ensemble. Free enzyme adopted a conformation in which the proximal pair was closer together and therefore the S2-S1 cleft was narrower than in the cathepsin K/substrate complex. The distal pair was distributed between two distinct populations which did not deviate considerably from the distances determined for the cathepsin K/substrate complex. In the presence of the inhibitors NSC13345 and NSC94914, multiple populations were observed, mirroring the increased flexibility of cathepsin K in the presence of these effectors (Fig 4). Distances determined for the former were overall similar to free enzyme, whereas the latter also induced an additional population with a significantly narrower S2-S1 cleft, akin to cluster 3 in Fig 4E. In contrast, C4S stabilized conformations with interatomic distances between each pair similar or greater than in the cathepsin K/substrate complex regardless of the site to which C4S was bound.

**Table 1:** Distribution of interatomic distances between residues lining the S1 and S2 sites in cathepsin K in MD simulations and in X-ray structures. All distances are given as mean values (± SD) calculated assuming normal distribution of the values. Where multiple distinct populations were observed, ratios between population sizes are given in parentheses.

	Proximal pair		Distal pair
	65Cα-161O	65Cα-161Cβ	66O-160O
Cathepsin K (X-Ray)	7.5 ± 0.2	9.6 ± 0.3	8.3 ± 0.5
Cathepsin K	6.2 ± 0.6	7.5 ± 1.0	7.1 ± 0.5
			8.0 ± 1.2
			(1: 1.7)
Cathepsin K/Substrate	7.6 ± 0.2	9.5 ± 0.4	7.6 ± 0.4
Cathepsin K/NSC13345	6.2 ± 0.6	7.3 ± 0.7	6.8 ± 0.5
		9.2 ± 0.6	7.8 ± 1.0
		(4: 1)	(1.2: 1)
Cathepsin K/NSC94914	4.0 ± 0.4	4.9 ± 0.5	4.3 ± 0.3
	6.2 ± 0.8	7.1 ± 0.7	7.1 ± 1.5
	7.9 ± 0.6	9.3 ± 0.7	(1: 12)
	(1.5: 2.8: 1)	(1: 1.2: 1.1)	
Cathepsin K/C4S(1)	7.3 ± 0.7	6.9 ± 0.9	8.3 ± 0.7
		9.2 ± 0.6	
		(1: 5.1)	
Cathepsin K/C4S(2)	7.5 ± 0.8	9.0 ± 0.7	8.3 ± 0.7
Cathepsin K/C4S(3)	7.5 ± 0.7	7.8 ± 0.8	9.6 ± 0.9

**Fig 6:**
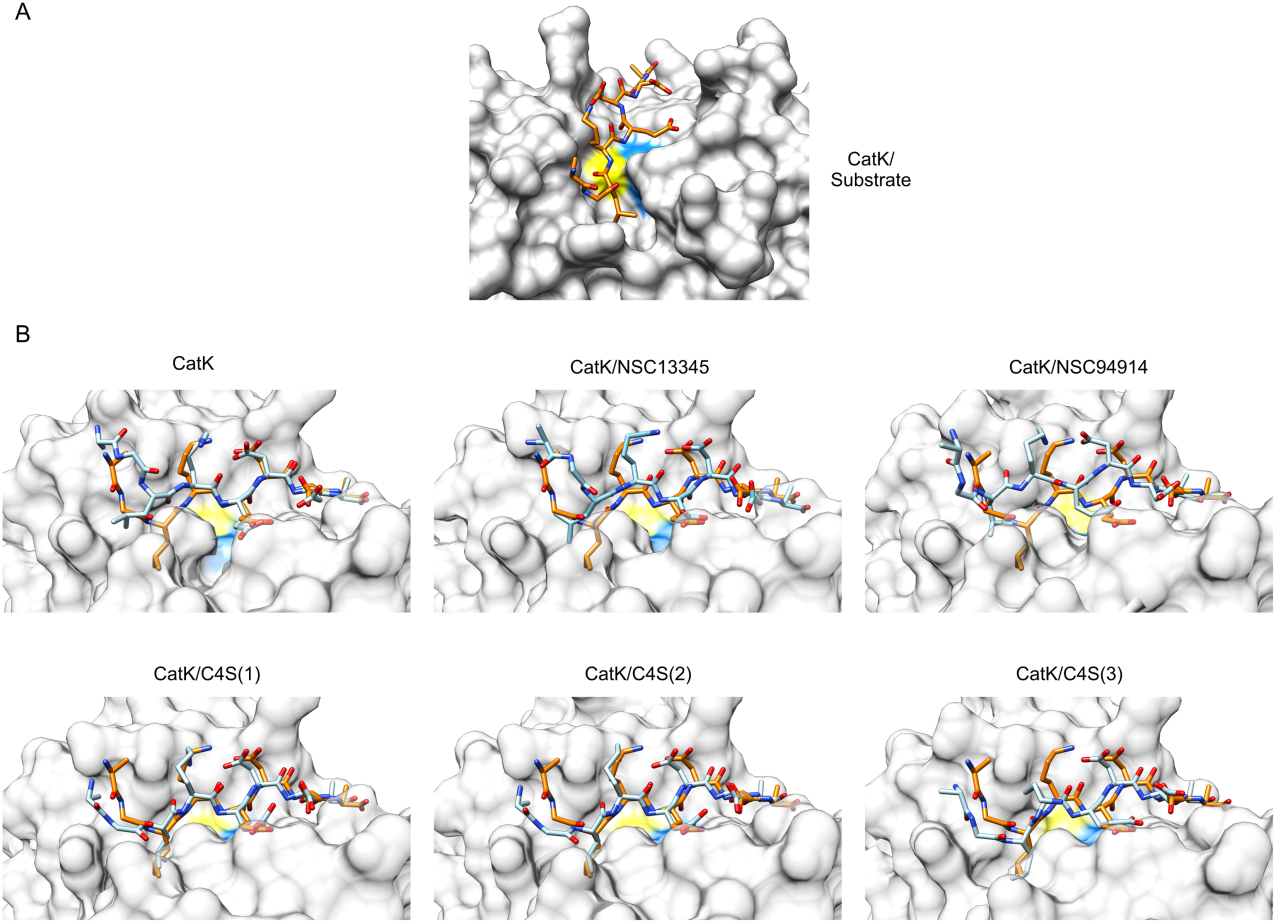
Docking of the substrate AGLKEDDA into the active sites of average MD conformers of cathepsin K. (A) Best docking solution of the substrate AGLKEDDA (orange-themed sticks) bound to the average MD conformer from the simulation of the enzyme/substrate complex which illustrates productive binding of the substrate into the active site. (B) Best solutions of docking of AGLKEDDA to the average MD conformers from simulations of free cathepsin K, in complexes with small molecule effectors NSC13345 and NSC94914, and in complexes with C4S bound to each of the three known binding sites, respectively. The enzyme is shown in a transparent surface representation. The docked substrate is shown as blue-themed sticks. For comparison, the productively bound substrate molecule from panel A (orange-themed sticks) was superposed onto each complex. In both panels, the positions of active site Cys and His residues are colored yellow and blue, respectively. Molecular graphics were prepared with UCSF CHIMERA software [36].

Experimental data had collectively shown, that all effectors predominantly affect *K*_m_ with only minor variations observed in the catalytical rates of the enzyme [10, 15, 17, 24]. To assess whether the observed differences in the shape of S2-S1 cleft suffice to affect the substrate affinity of cathepsin K, we docked the substrate AGLKEDDA into the active sites of average MD conformers calculated from each simulation. It must be noted that cathepsin K was treated as a rigid molecule, therefore the effects of induced fit of the substrate were not taken into account in the docking experiments. The pose of AGLKEDDA bound in a substrate-like manner is shown in Fig 6A. It was produced by re-docking the substrate to the average MD conformer of cathepsin K from the simulation of the cathepsin K/substrate complex. Free cathepsin K adopted a narrower conformation in the S2-S1 cleft, as discussed above. As a consequence, docking experiments produced binding poses in which the non-primed part of the substrate failed to bind into the active site in a substrate-like manner (Fig 6B). Most notably, Leu2 of the substrate was prevented from binding into the S2 pocket. Taking into account that docking was performed with a rigid receptor molecule, these results support the binding of substrate to the enzyme via an induced fit mechanism where the non-primed sites of the enzyme must move further apart in order to accomodate the substrate. We have, in fact, previously observed two separate, catalytically competent, conformations of cathepsin K at neutral pH *in vitro* [10], which appear to be analogous to the conformations observed in the computational experiments herein. The tense (T) state, akin to the conformation of free cathepsin K herein, was stabilized by low salt buffers in the absence of substrate, had lower substrate affinity and was less susceptible to inhibition by irreversible and reversible inhibitors, whereas the relaxed (R) state with higher substrate affinity was stabilized by high salt concentration, similar to the conditions used to produce protein crystals. Moreover, a slow hysteretic transition from the T to the R state was observed when the enzyme was stimulated with low concentrations of substrate. The structural basis for the experimentally observed transitions thus appears to be the herein identified conformational change involving the non-primed sites of the enzyme.

In a similar fashion, docking of the substrate AGLKEDDA to average MD conformers of cathepsin K in complexes with allosteric modifiers was performed and analyzed (Fig 6B). Small molecule effectors NSC13345 and NSC94914 stabilized conformations with a narrow S2-S1 cleft similar to the free enzyme. As a result the substrate failed to bind into the S2 pocket. Both effectors can thus be predicted to stabilize the T state. This is in agreement with experimental data which have shown that both effectors act predominantly by reducing the substrate affinity of cathepsin K [15, 17, 24]. In contrast, C4S stabilized conformations with a wider S2-S1 cleft. Accordingly, the substrate could be readily docked into the active site in a manner analogous to the cathepsin K/substrate complex. This indicates that C4S stabilizes the R state, as observed experimentally at physiological plasma pH [10].

### Other cathepsins

Based on the data presented in the previous sections, we also sought to examine the variability of other human cathepsin endopeptidases (cathepsins L, V, S and F) and its potential effects on their activity. Unfortunately, no allosteric effectors of family members other than cathepsin K have yet been characterized at the structural level. However, there have, been several indications of their existence. GAGs, in particular, have been shown to modulate the activity and stability of not only cathepsin K but also of cathepsins S and B [28, 29] as well as several non-animal endopeptidases including papain [30], cruzipain from *Trypanosoma cruzi* [31], rhodesain from *Trypanosoma brucei* [32] and cysteine peptidase B from *Leishmania mexicana* [33]. Moreover, DNA has been shown to act in a fashion similar to GAGs and regulate the susceptibility of cathepsin V towards inhibition by serpins [34]. Identification of mechanisms of active site adaptation similar to cathepsin K would indicate that papain-like peptidases share a common mechanism of allosteric regulation that is exploited by GAGs and could potentially also be targeted for drug design.

**Fig 7:**
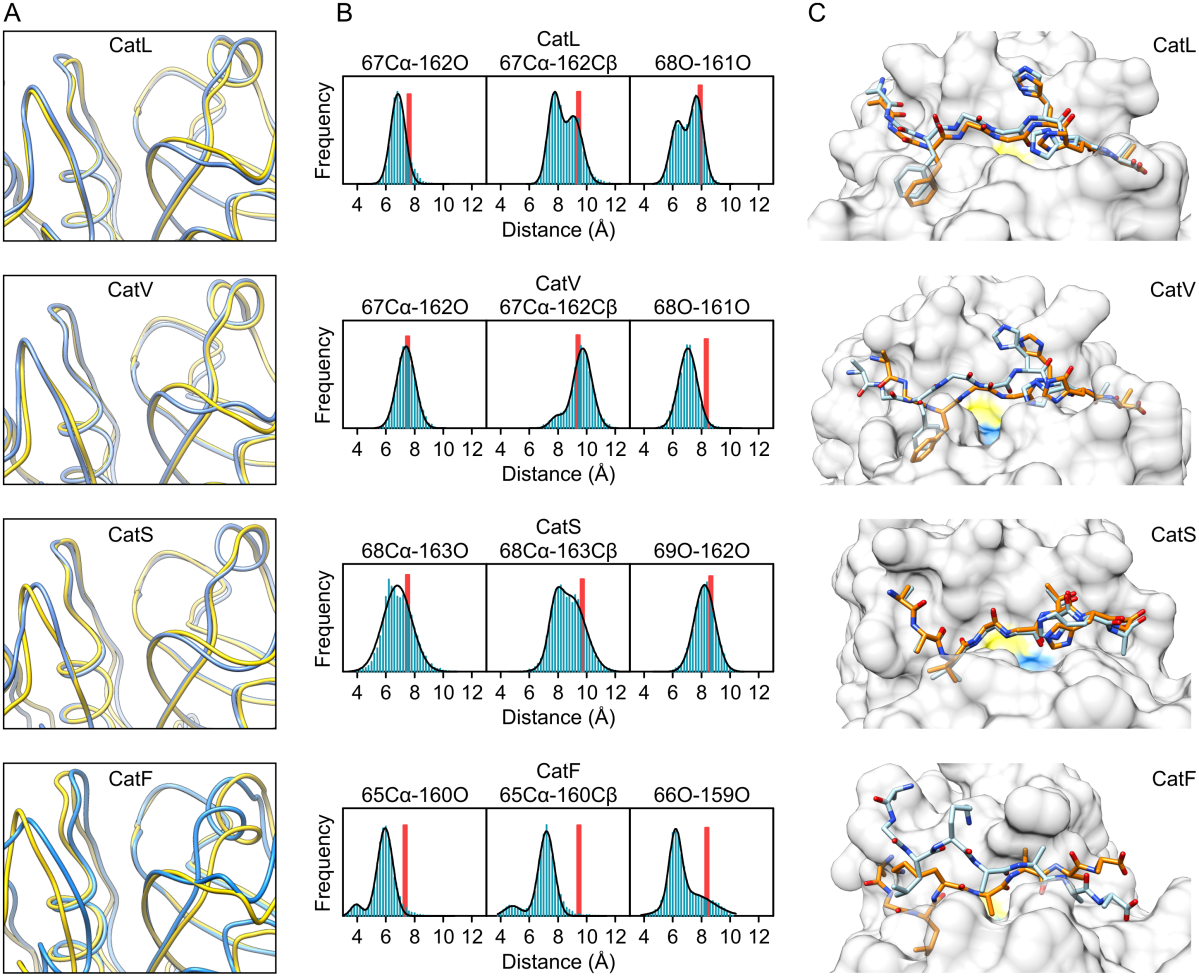
Conformational variability of the active sites of other human papain-like endopeptidases. (A) Superpositions of initial X-ray structures (yellow ribbons) and average MD conformers (blue ribbons) of human cathepsins L, V, S and F (PDB accession codes 1MHW, 1FH0, 2OP3 and 1M6D, respectively). (B) Interatomic distances between residues lining the S2-S1 cleft in each peptidase. All populations were segregated into 0.2 Å wide bins. The curves were fitted assuming normal distribution of interatomic distances with models assuming one or two populations, as appropriate. Distances observed in initial X-ray structures are represented by red bars. The analyses were performed with Graph Pad Prism 5.0 Software. (C) Best solutions of docking substrate molecules into the active sites of cathepsins L, V, S and F. The proteins are shown in surface representation and the positions of catalytic Cys and His residues are colored yellow and blue, respectively. For each enzyme, solutions obtained with the initial X-ray structure and the average MD conformer are shown in superposition and the docked substrates are colored in orange and blue themes, respectively. The substrates used were AGFGGHHA for cathepsins L and V, AAVGGTHA for cathepsin S and AGLKAAAA for cathepsin F. Molecular graphics were prepared with UCSF CHIMERA software [36].

As above, we perfomed MD simulations followed by analysis of interatomic distances and docking of substrate molecules to X-ray and average MD structures. Appropriate substrates for each enzyme were constructed according to their specificity profiles [26, 27, 35]. The examined endopeptidases showed varying extent of conformational change in the loops lining the non-primed sites (Fig 7A). The width of the S2-S1 cleft was determined from the distributions of interatomic distances between the proximal and distal atom pairs equivalent to those in cathepsin K. The results are shown graphically in Fig 7B and the numerical values are given in Table 2. Similar to cathepsin K, all free enyzmes showed tendencies towards a narrower S2-S1 cleft in comparison to initial X-ray structures. The effect was most apparent in cathepsin F and could be attributed predominantly to conformational changes in loop 1. In contrast, cathepsins L, V and S showed conformational changes predominantly in loop 3. Judging from the superposition of X-ray and average MD conformers, cathepsin S exhibited the least conformational change. It did, however, exhibit the widest interval of proximal pair distances, indicating that the S2-S1 cleft in cathepsin S is somewhat more flexible than in its homologs.

**Table 2:** Distribution of interatomic distances between residues lining the S1 and S2 sites in cathepsin L, V, S and F in MD simulations. All distances are given as mean values (± SD) calculated assuming normal distribution. Where multiple distinct populations were observed, ratios between population sizes are given in parentheses.

	Proximal pair		Distal pair
Cathepsin L	67Cα-162O	67Cα-162Cβ	68O-161O
	7.5 ± 0.2	9.6 ± 0.3	8.3 ± 0.5
Cathepsin V	67Cα-162O	67Cα-162Cβ	68O-161O
	7.4 ± 0.6	7.9 ± 0.5	7.1 ± 0.6
		9.7 ± 0.6	
		(1: 10)	
Cathepsin S	68Cα-163O	68Cα-163Cβ	69O-162O
	6.8 ± 1.0	7.8 ± 0.4	8.2 ± 0.7
		8.9 ± 1.0	
		(1: 4.2)	
Cathepsin F	65Cα-160O	65Cα-160Cβ	66O-159O
	3.9 ± 0.4	4.8 ± 0.6	6.2 ± 0.4
	6.0 ± 0.5	7.2 ± 0.5	7.4 ± 1.4
	(1: 11)	(1: 8)	(1: 1)

These observations were mirrored in the results of docking experiments in which we compared the poses of substrate molecules docked into the active sites of X-ray and average MD conformers of each peptidase, where X-ray conformers represent the bound state, since they were derived from X-ray structures of enzyme/inhibitor complexes, and average MD conformers represent the free state. The results are shown in Fig 7C. The docking poses of the substrate AAVGGTHA bound to cathepsin S were virtually identical, as expected due to the lack of observed conformational change. Less expectedly, similar docking poses were also obtained for the substrate AGFGGHHA bound to cathepsin L, indicating that narrowing of the S2-S1 cleft does not necessarily adversely affect substrate affinity. In contrast, cathepsins V and F, which had average MD structures with pronouncedly tighter S2-S1 clefts akin to cathepsin K (see Fig 6 for comparison), exhibited similar steric hindrance in adopting their respective substrates (AGFGGHHA and AGLKAAAA, respectively) into the active site cleft. Taken together, these results indicate that other papain-like endopeptidases exhibit similar active site dynamics as cathepsin K and thus may be regulated allosterically in a similar manner.

## Conclusion

Allosteric regulation is slowly surfacing as a common regulatory strategy in papain-like peptidases. Our understanding at the structural level has so far been restricted to the identity of allosteric sites and a limited number of effectors, whereas knowledge on how these effectors work has been limited due to the absence of obvious conformational change observable in X-ray structures. Herein we have made significant progress in our comprehension of this mode of action. We now have strong indications that allosteric regulation in cathepsin K, the primarily investigated papain-like peptidase from the allosteric perspective, proceeds via stabilization of conformational states with different accesibility of the S2 site, the primary specificity determinant, to the substrates. Conformational plasticity is an inherent property of cathepsin K and our data indicate that similar mechanisms also operate in other cysteine cathepsin peptidases. Altogether this analysis provides an important basis for experimental investigation of allostery in this family.

## Methods

### Structural data collection and visualization

All coordinates used for analyses and simulations were retrieved from the RCSB Protein Data Bank (www.rcsb.org). A complete list of entries used in this work is given in S1 Table. UCSF Chimera [36] was used for superposition and other coordinate manipulations, as well as for visualization of all molecular structures presented in the manuscript.

### MD simulations

MD simulations were performed using NAMD [37] version 2.11 with CUDA acceleration on a single node-desktop computer equipped with a quad-core processor and a CUDA-capable graphics card. The starting coordinates for all simulations were retrieved from the RCSB Protein Data Bank (PDB) under the following accession numbers: 1ATK and 1U9V for human cathepsin K, 3C9E and 4N8W for human cathepsin K in complexes with C4S, 5J94 for human cathepsin K mutant Cys25Ser in complex with NSC13345, 5JA7 for human cathepsin K mutant Cys25Ser in complex with NSC94914, 1MHW for human cathepsin L, 1FH0 for human cathepsin V, 2OP3 for human cathepsin S and 1M6D for human cathepsin F, respectively. CHARMM 27 force field parameters were used for the proteins [38] and the ligands were parametrized using the SwissParam web server [39]. The simulations were run in explicit solvent under periodic boundary conditions at a constant temperature of 298 K and with default values of interaction parameters. The systems were minimized for 200 steps and equilibrated for 200 ps. The trajectories collected at a frequency of 10 ps per frame. The lenghts of individual runs were between 20 ns and 150 ns. In the case of ligand dissociation from the binding site, which occured occasionally with all ligands, only frames containing ligand bound to the binding site were retained. Final trajectories were pooled from three runs. Total simulation times collected were 200 ns for ligand-free cathepsin K, 100 ns for cathepsin K/substrate and cathepsin K/C4S complexes, and for cathepsins L, V, S and F, 125 ns for the cathepsin K/NSC94914 complex and 240 ns for the cathepsin K/NSC13345 complex.

The Wordom program [40] was used to calculate average structures (average MD conformers)from the trajectories, to calculate RMSF and per-frame distances between designated atom pairs, as described in the text, and to cluster the trajectories of simulations of cathepsin K/NSC13345 and cathepsin K/NSC94914 complexes. Clustering was performed using the quality threshold (qt) method and root mean square distances at a cut-off radius of 1.2 Å

### Principal component analysis

PCA was performed with the Bio3D package [41, 42] on the ensemble of structures collected in S1 Table. If necessary, the PDB files were manually edited to replace non-standard residue codes (e.g. derivatives of the active site Cys25 residue) with standard three-letter codes. The sequences were aligned using the *pdbaln* command and the alignment then manually refined (supplied as S2 Dataset). The ensemble was filtered to remove conformationally redundant structures using the *filter*.*rmsd* command with a cut-off value of 0.1 Å. Based on the alignment, the invariant protein core was determined using the *core*.*find* command, all sequences superposed onto the core and then analyzed with PCA. The *mktrj* command was used to create trajectories representing the conformational variability along each principal component, which were analyzed using UCSF Chimera [36]. The *rmsf* command was used to calculate the RMSF of the ensemble after its superposition onto the invariant core. Trajectories from MD simulations described in the previous section were superposed onto the invariant core with the *fit*.*xyz* command and then each frame projected onto the principal components of the X-ray ensemble using the *project*.*pca* command.

### Normal mode analysis

Normal mode analysis was performed using the Bio3d package [41, 42] on an ensemble of non-redundant 55 structures of cathepsin K. The ensemble was obtained after filtering the sample of all cathepsin K structures (S1 Table) with the *filter*.*rmsd* command using a cut-off value of 0.1 Å to remove conformationally redundant strutures. The first five normal modes were converted to trajectories with the *mktrj* command and visualized in UCSF Chimera [36].

### Docking of the substrate AGLKEDDA into the active site of cathepsin K

As the ligand molecule, the octapeptide Ala-Gly-Leu-Lys-Glu-Asp-Asp-Ala (AGLKEDDA) was constructed *de novo* using UCSF Chimera [36] and the torsion angles constrained to an extended parallel β-strand conformation. The molecule was then prepared for docking using AutoDock Tools [43]. Polar hydrogens were added to the molecule, all single bonds defined as rotatable and peptide bonds designated as non-rotatable. As the receptor, the cathepsin K structure retrieved from the Protein Data Bank under accession number 1ATK was used. All non-protein atoms were removed and non-polar hydrogens added. AutoDock Vina [44] was used to dock the ligand into the active site of the receptor, which was treated as a rigid body. 20 docked poses were calculated at an exhaustiveness level of 20. Model selection was performed manually by screening solutions according to known data on the binding of substrates into the active site of papain-like endopeptidases (see refs. [19, 45, 46]), i.e. extended conformation of the peptide chain with all residues interacting with the enzyme, residue Leu2 bound in the S2 pocket and the scissile bond between Lys3 and Asp4 positioned above the catalytic residue Cys25.

The same procedure was used to dock the substrate AGLKEDDA into the active sites of average MD conformers of cathepsin K derived from MD trajectories. Since structure averaging over MD trajectories results in distortion of flexible side chains, correct geometry of the receptors was first restored by energy minimization with NAMD [37]. Minimization was performed until a negative total energy of the protein was reached, which was between 200 and 300 steps. Superposition of the minimized and initial structures showed that the minimization did not cause significant changes in the coordinates of backbone atoms.

### Docking of substrate molecules to other papain-like endopeptidases

Other substrate molecules were docked into the active site of respective enzymes by the same procedure described in the previous section. The constructed substrates were Ala-Gly-Phe-Gly-Gly-His-His-Ala (AGFGGHHA) for cathepsins L and V, Ala-Ala-Val-Gly-Gly-Thr-His-Ala (AAVGGTHA) for cathepsin S and Ala-Gly-Leu-Lys-Ala-Ala-Ala-Ala (AGLKAAAA) for cathepsin F. The PDB entries used to retrieve the macromolecular coordinates were 1MHW, 1FH0, 2OP3 and 1M6D for cathepsins L, V, S and F, respectively. As above, docking was performed using the initial X-ray conformers and average structures calculated from MD trajectories.

## Supporting information

S1 Fig Representative members of the papain-like family. The conserved core is colored blue and conformationally variable regions discussed in the manuscript are colored dark yellow. The remaining part of the molecules is colored tan. PDB entries used in the representations are 1ATK for human cathepsin K, 1NB3 for human cathepsin H, 4YYQ for the fig protease ficin, 1YVB for falcipain-2, the major cysteine protease of Plasmodium falciparum, 3OIS for xyllelain from Xyllela fastidiosa, 4D59 for Cwp84 from Clostridium difficile, 1CSB for human cathepsin B, 1EF7 for human cathepsin X and 1K3B for dipeptidyl-peptidase I. The graphics were prepared with UCSF Chimera Software.
(PDF)

S2 Fig Pairwise superpositions of human cathepsin K (tan) and other representative papain-like peptidases (blue). The superpositions were constructed from the PDB entries shown individually in S1 Fig. The graphics were prepared with UCSF Chimera Software.
(PDF)

S3 Fig Normal mode analysis of cathepsin K. Normal modes 1 through 5 (from top to bottom) calculated for an ensebmle of 55 non-reduntant cathepsin K structures. The representation contains only non-gapped positions in the sequence alignment (residues 3 through 215). The analysis was performed with the Bio3d package and the graphics prepared with UCSF Chimera Software.
(PDF)

S1 Table Protein Data Bank accession codes of entries used in this work. (PDF)

S1 Dataset The results of normal mode analysis in PDB format. (ZIP)

S2 Dataset Multiple sequence alignment used in this work in FASTA format. (FA)
