# Supplementary material for: Computational investigation of conformational variability and allostery in cathepsin K and other related peptidases
Source: PLoS One. 2017 Aug 3;12(8):e0182387. doi: 10.1371/journal.pone.0182387 (PMC5542433; doi:10.1371/journal.pone.0182387)
Supplement: S1 Fig — The conserved core is colored blue and conformationally variable regions discussed in the manuscript are colored dark yellow. The remaining part of the molecules is colored tan. PDB entries used in the representations are 1ATK for human cathepsin K, 1NB3 for human cathepsin H, 4YYQ for the fig protease ficin, 1YVB for falcipain-2, the major cysteine protease of Plasmodium falciparum, 3OIS for xyllelain from Xyllela fastidiosa, 4D59 for Cwp84 from Clostridium difficile, 1CSB for human cathepsin B, 1EF7 for human cathepsin X and 1K3B for dipeptidyl-peptidase I. The graphics were prepared with UCSF Chimera Software. (PDF) [file pone.0182387.s001.pdf]

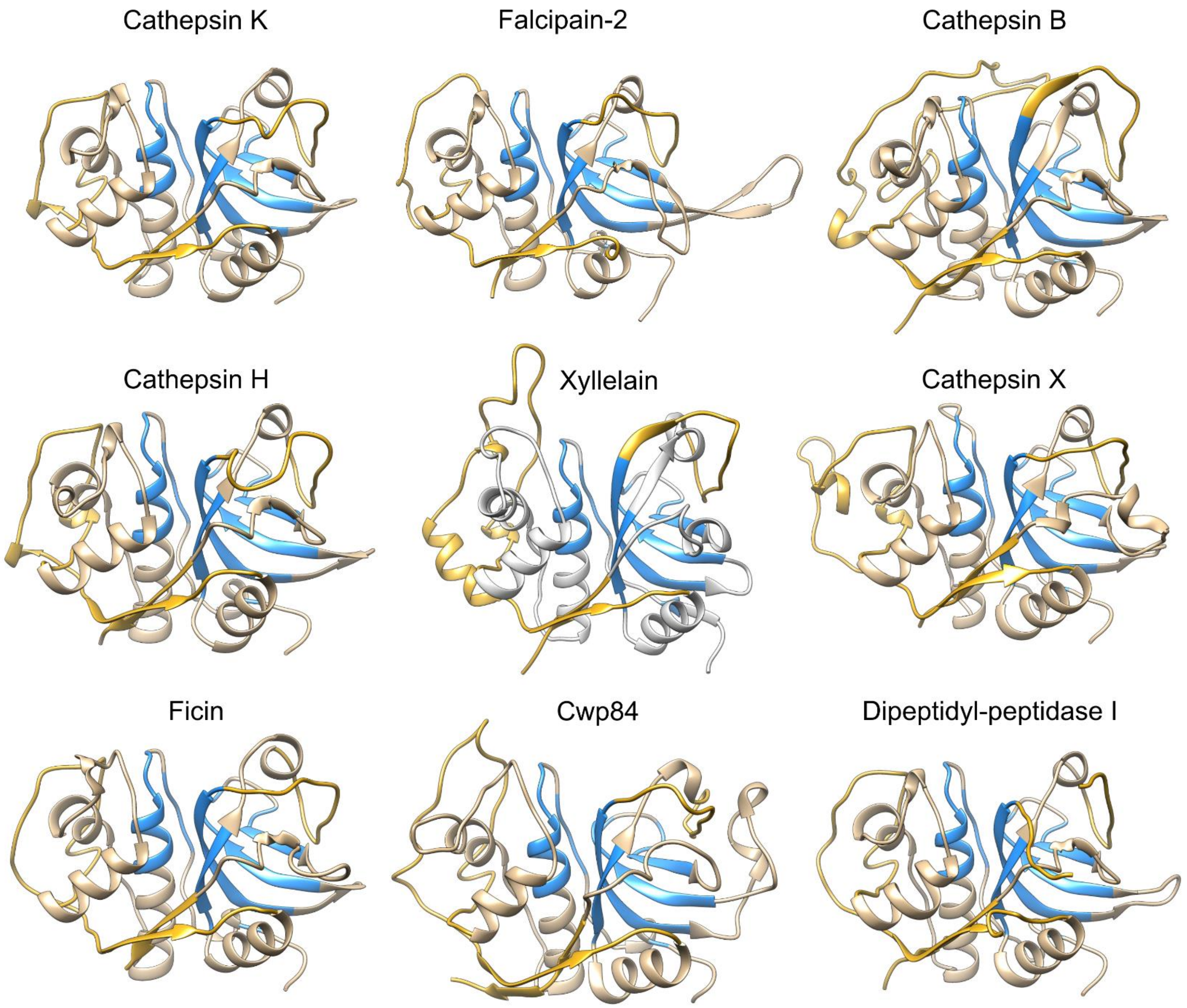

S1 Fig. Representative members of the papain-like family. The conserved core is colored blue and conformationally variable regions discussed in the manuscript are colored dark yellow. The remaining part of the molecules is colored tan. PDB entries used in the representations are 1ATK for human cathepsin K, 1NB3 for human cathepsin H, 4YYQ for the fig protease ficin, 1YVB for falcipain-2, the major cysteine protease of *Plasmodium falciparum*, 3OIS for xyllelain from *Xyllela fastidiosa*, 4D59 for Cwp84 from *Clostridium difficile*, 1CSB for human cathepsin B, 1EF7 for human cathepsin X and 1K3B for dipeptidyl-peptidase I. The graphics were prepared with UCSF Chimera Software.
