# Supplementary material for: Computational investigation of conformational variability and allostery in cathepsin K and other related peptidases
Source: PLoS One. 2017 Aug 3;12(8):e0182387. doi: 10.1371/journal.pone.0182387 (PMC5542433; doi:10.1371/journal.pone.0182387)
Supplement: S2 Fig — The superpositions were constructed from the PDB entries shown individually in S1 Fig. The graphics were prepared with UCSF Chimera Software. (PDF) [file pone.0182387.s002.pdf]

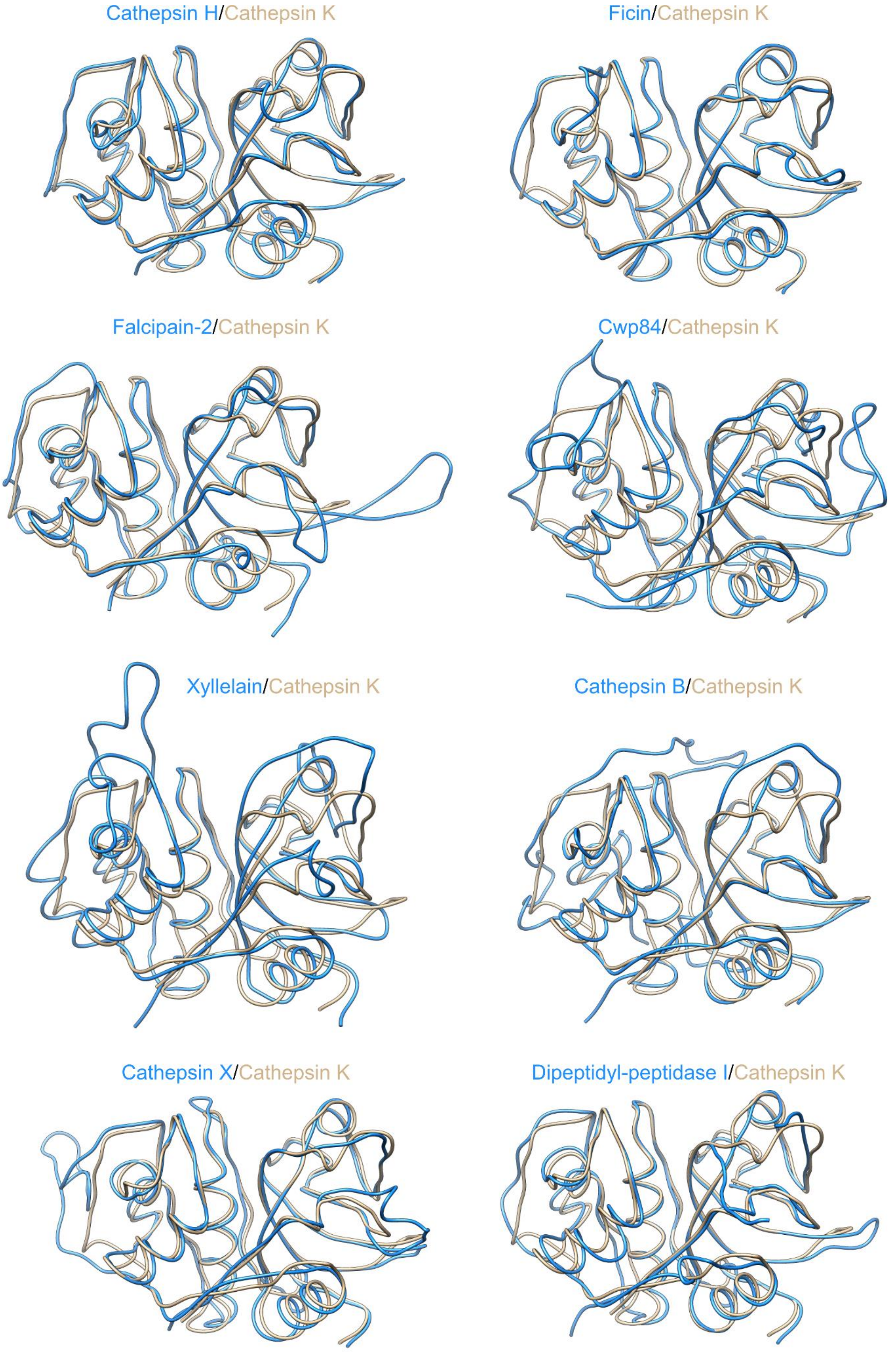

S2 Fig. Pairwise superpositions of human cathepsin K (tan) and other representative papain-like peptidases (blue). The superpositions were constructed from the PDB entries shown individually in Supplementary Figure S1. The graphics were prepared with UCSF Chimera Software.
