# Supplementary material for: Computational investigation of conformational variability and allostery in cathepsin K and other related peptidases
Source: PLoS One. 2017 Aug 3;12(8):e0182387. doi: 10.1371/journal.pone.0182387 (PMC5542433; doi:10.1371/journal.pone.0182387)
Supplement: S3 Fig — Normal modes 1 through 5 (from top to bottom) calculated for an ensebmle of 55 non-reduntant cathepsin K structures. The representation contains only non-gapped positions in the sequence alignment (residues 3 through 215). The analysis was performed with the Bio3d package and the graphics prepared with UCSF Chimera Software. (PDF) [file pone.0182387.s003.pdf]

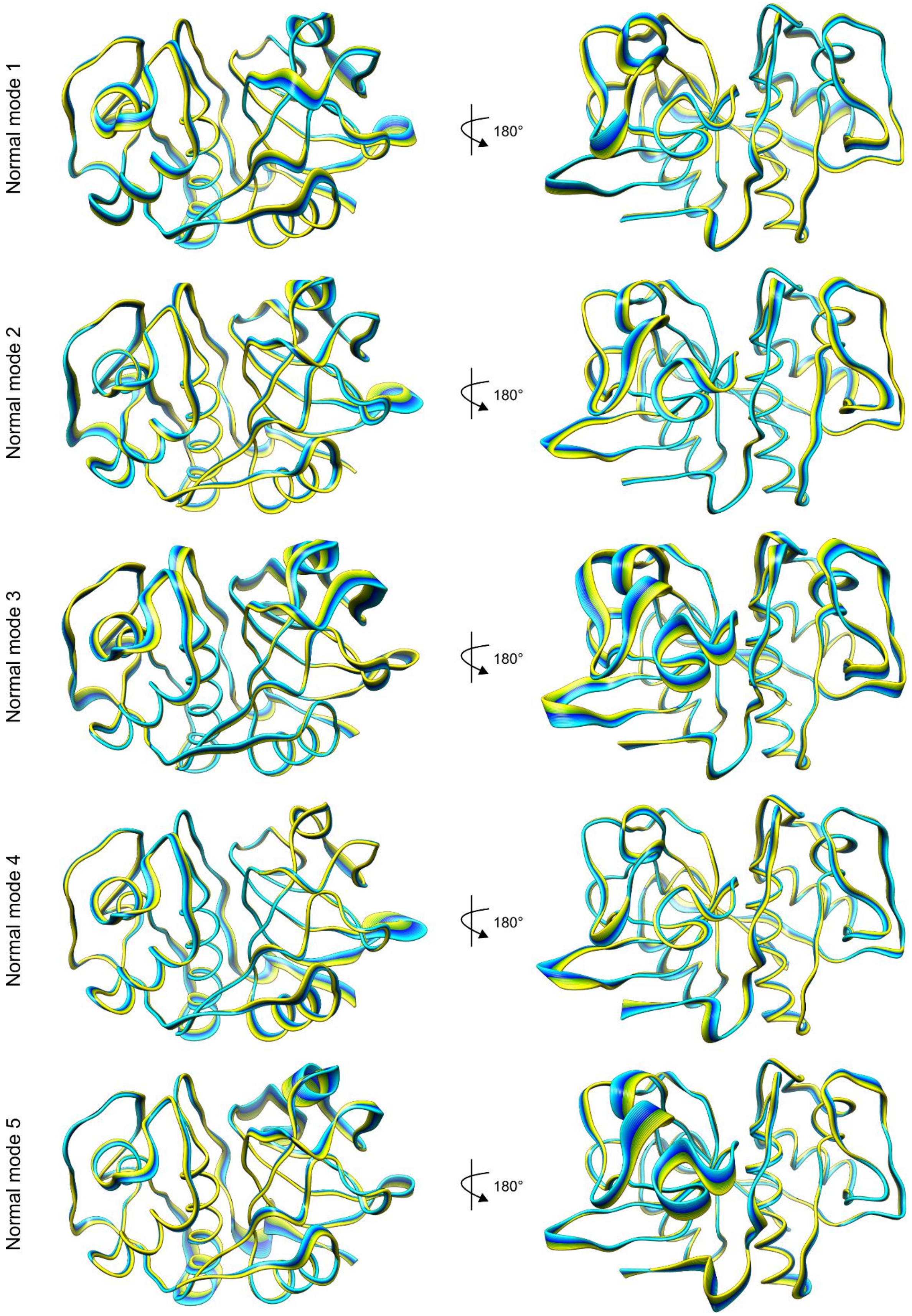

S3 Fig. Normal mode analysis of cathepsin K. Normal modes 1 through 5 (from top to bottom) calculated for an ensebmle of 55 non-reduntant cathepsin K structures. The representation contains only non-gapped positions in the sequence alignment (residues 3 through 215). The analysis was performed with the Bio3d package and the graphics prepared with UCSF Chimera Software.
