# Supplementary material for: Computational investigation of conformational variability and allostery in cathepsin K and other related peptidases
Source: PLoS One. 2017 Aug 3;12(8):e0182387. doi: 10.1371/journal.pone.0182387 (PMC5542433; doi:10.1371/journal.pone.0182387)
Supplement: S1 Table — (PDF) [file pone.0182387.s004.pdf]

S1 Table. Protein Data Bank accession codes of entries used in this work.

**Cathepsin K**	**Cathepsin L**	3IEJ	2DCC	1KHQ	1EWP
1ATK	1CJL	3KWN	2DCB	1KHP	1AIM
1AU0	1CS8	3MPF	2DCA	2CIO	2AIM
1AU2	1ICF	3MPE	2DC9	3E1Z	1YVB
1AU3	1MHW	3N3G	2DC8	3U8E	2GHU
1AU4	2NQD	3OVZ	2DC7	2PRE	3PNR
1AYU	2XU1	3OVX	2DC6	3BCN	3BWK
1AYV	2XU3	3N4C	2IPP	2PNS	3BPF
1AYW	2XU4	4BSQ	3CBJ	2BDZ	3BPM
1BGO	2XU5	4BS5	3CBK	1CQD	2OUL
1BY8	2YJB	4BPV	3K9M	1MEG	
1MEM	2YJC	4BS6	3AI8	3IQQ	**Bacteria**
1NL6	2YJ2	4BQV	4HWY	1YAL	3OIS
1NLJ	2YJ8	4MZO	4N4Z	4YYQ	4D59
1Q6K	2YJ9	4MZS	5FPW	4YYR	4D5A
1SNK	3BC3	4P6E		4YYS	4CI7
1TU6	3HHA	4P6G	**Cathepsin X**	4YYU	
1U9V	3HWN		1DEU	4YYV	
1U9W	3H8B	**Cathepsin V**	1EF7	4YYW	
1U9X	3H8C	1FH0		1GEC	
1VSN	3H89	3H6S	**Cathepsin C**	3P5U	
1YK7	3K24	3KFQ	1JQP	3P5V	
1YK8	3IV2		1K3B	3P5W	
1YT7	3KSE	**Cathepsin L**	2DJF	3P5X	
2ATO	3OF8	**non-vertebrate**	2DJG	1AEC	
2AUX	3OF9	2O6X	3PDF	2ACT	
2AUZ	4AXL	3F75	4CDC		
2BDL	4AXM	3QJ3	4CDD	**Unicellular**	
2F7D	5F02	3QT4	4CDE	**eukaryotes**	
2FTD			4CDF	2P86	
2R6N	**Cathepsin S**	**Cathepsin F**	4OEL	2P7U	
3C9E	1GLO	1M6D	4OEM	3IUT	
3H7D	1NQC			1EWL	
3KW9	1NPZ	**Cathepsin H**	**Plant**	4QH6	
3KWB	1MS6	1NB3	**Endopeptidases**	4W5C	
3KWZ	2FQ9	1NB5	1PPD	4XUI	
3KX1	2G6D	8PCH	9PAP	4W5B	
3O0U	2F1G		1PE6	4PI3	
3O1G	2FT2	**Cathepsin B**	1POP	4KLB	
3OVZ	2FRQ	1HUC	1PIP	3LXS	
4DMX	2FRA	1CTE	1STF	3KKU	
4DMY	2FUD	1CPJ	1PPP	3I06	
4N8W	2FYE	1THE	1PPN	3HD3	
4N79	2HH5	1CSB	3IMA	2OZ2	
4X6H	2G7Y	1MIR	3TNX	1U9Q	
4X6I	2HXZ	1PBH	3USV	1EWM	
4X6J	2H7J	2PBH	4KP9	1EWO	
5J94	2C0Y	3PBHJ	4QRX	1ME3	
5JA7	2HHN	1QDQ	4QRG	1ME4	
5JH3	2OP3	1GMY	4QRV	1F29	
7PCK	2R9M	1ITO	1BP4	1F2A	
	2R9N	1SP4	1BQI	1F2B	
	2R9O	2DCD	1CVZ	1F2C	
